# Cumulative Violence and Post-Traumatic Stress: An Integrative Model of Coping and Resilience Among Women Exposed to Sexual and Conflict-Related Violence

**DOI:** 10.3390/ejihpe15060110

**Published:** 2025-06-13

**Authors:** Naama Bar, Stav Shapira, Orna Braun-Lewensohn

**Affiliations:** 1 Conflict Management & Resolution Program, Ben-Gurion University of the Negev, Beer-Sheva 8410501, Israelornabl@bgu.ac.il (O.B.-L.); 2 School of Public Health, Faculty of Health Sciences, Ben-Gurion University of the Negev, Beer-Sheva 8410501, Israel

**Keywords:** sexual violence, conflict-related violence, intersection of exposures, resilience, coping strategies, post-traumatic symptoms

## Abstract

This study explored how exposure to sexual and conflict-related violence relates to the severity of post-traumatic symptoms and how personal and community resilience factors and coping strategies mediate that relationship. Self-report questionnaires were completed by 568 Israeli women, who were classified into four exposure groups: (a) high sexual violence, (b) high conflict-related violence, (c) dual high exposure, and (d) low exposure. Significant differences were found between the group exposed solely to conflict-related violence and the groups exposed to sexual or both kinds of violence. Those who had been exposed to both types of violence reported lower levels of a personal sense of coherence, greater use of non-adaptive coping strategies, and more severe post-traumatic symptoms, as compared to the high conflict-related violence group and the low-exposure group. The dual-high-exposure group also reported lower levels of community resilience than the high-conflict-related-violence group and less use of adaptive coping strategies than the high-sexual-violence group. The severity of post-traumatic symptoms was explained by combined exposure to both sexual and conflict-related violence, personal resilience, and the use of non-adaptive coping strategies. These findings emphasize the unique psychological burden associated with intersecting exposures.

## 1. Introduction

The psychological toll of prolonged exposure to conflict-related violence on civilian populations has been widely documented (Greene et al., 2018; Pat-Horenczyk & Schiff, 2019; Shapira et al., 2020; Stein et al., 2018). For individuals with a history of personal trauma—particularly sexual violence—renewed exposure to mass violence can exacerbate distress by reactivating traumatic memories and destabilizing core assumptions about safety and trust (Chung & Freh, 2019; Herman, 1992; Punamäki et al., 2019). Sexual violence, which often undermines one’s basic sense of bodily integrity and relational security, may interact with the threat of conflict to produce compounded psychological harm.

The ongoing conflict between Israel and Gaza has exposed residents of the area bordering Gaza to frequent security threats, such as rocket fire, sirens, and infiltrations. On 7 October 2023, Hamas carried out an unprecedented terrorist attack on Israeli communities near the Gaza border, involving mass killings, infiltrations, and abductions. This led to the outbreak of the war known in Israel as “Iron Swords”. Since the outbreak of the war, survivors of sexual violence have reported heightened post-traumatic symptoms, including hyperarousal, intrusive memories, and heightened avoidance (Kamin et al., 2023). For many, war-related stressors serve not only as new traumatic events, but also as triggers that reactivate unresolved sexual trauma (Ben Eliyahu et al., 2024).

These dynamics underscore the need to better understand how intersecting and cumulative exposures to different forms of violence shape mental health outcomes, as well as how individuals draw on resilience resources and coping strategies to manage such complex stress. Despite emerging literature on intersectional trauma and gendered violence in conflict zones (e.g., Ainamani et al., 2020; Chudzicka-Czupała et al., 2023; Okeke-Ihejirika et al., 2018), few empirical studies test integrated, theory-driven models that examine how resilience and coping mechanisms operate in the face of compounding stressors.

This study, initiated prior to the current war, contributes to addressing this gap. It focuses on the intersection of sexual and conflict-related violence and how this dual exposure shapes psychological outcomes in women. The study draws on an integrative theoretical model combining the ecological approach, the interactional model of coping with stress, and the salutogenic theory. Using a sample of Israeli women, we examined which coping strategies and resilience factors mitigate post-traumatic symptoms across different exposure profiles.

Our goal was not only to explore the psychological consequences of intersecting violence, but also to identify protective mechanisms that may buffer these effects in chronically insecure environments. It is important to note that the present study was conducted during a relatively calm period in terms of conflict-related violence, prior to the 7 October terrorist attacks and the subsequent war. While this timing may limit the generalizability of the findings to acute wartime settings, it offers a valuable baseline for understanding how women with prior trauma histories cope under conditions of ongoing threat. As such, this study provides insight into resilience and vulnerability before escalation—insight that may inform early intervention strategies in future conflict situations.

### 1.1. Ongoing Exposure to Conflict-Related Violence

Ongoing exposure to warfare and terrorist events destabilizes an individual’s fundamental sense of security (Chung & Freh, 2019; Punamäki et al., 2019) and may lead to the development of post-traumatic symptoms (Greene et al., 2018). However, exposure to conflict-related violence does not necessarily result in psychological distress. In fact, studies indicate that most individuals exposed to such violence cope adaptively and do not develop significant psychological disorders (Zelis & Shapira, 2025). This suggests the existence of resilience mechanisms that influence the incidence and severity of pathological symptoms among individuals exposed to conflict-related violence (Greene et al., 2018; Stein et al., 2018).

### 1.2. Exposure to Sexual Violence

Globally, approximately one in three women report having experienced physical sexual violence (WHO, 2021), a prevalence reflected in Israeli data as well (Association of Rape Crisis Centers in Israel, 2022). Given this prevalence, it is likely that a substantial proportion of women living in areas heavily affected by the ongoing conflict, such as communities near the Israel–Gaza border, have experienced sexual violence.

Sexual assault, whether occurring in childhood or adulthood, has complex and long-lasting consequences, including physical, psychological, behavioral, social, and cognitive impacts that may persist long after the assault (Hailes et al., 2019; van der Kolk, 2014/2021). Although sexual trauma increases the risk of developing post-traumatic symptoms, studies indicate that some individuals who have experienced sexual violence maintain a high level of functioning. This heterogeneity is partially explained by the presence of various resilience factors, which play crucial roles in shaping individuals’ capacity to adapt and cope with sexual trauma (Domhardt et al., 2015).

### 1.3. Intersection of Conflict-Related and Sexual Violence

The term intersectionality, introduced by Kimberlé Crenshaw in 1989, describes how overlapping social conditions and experiences combine to produce effects greater than the sum of their individual parts (Cooper, 2015). When applied to stress and trauma, the intersection of acute and chronic stressors can generate a cumulative psycho-social burden, triggering neurological and physiological dysregulation (Nurius et al., 2013). Such cumulative exposure may erode coping resources, increasing vulnerability to psychological distress and psychopathology. Early-life adversities—such as childhood abuse—are particularly potent, heightening sensitivity to future threats. For example, Stevanović et al. (2016) found that early-life trauma heightened PTSD symptoms following war exposure, illustrating how stressors across life domains can compound one another.

Past sexual violence, particularly when experienced in childhood or within trusted relationships, can undermine basic assumptions about safety and relational trust (Herman, 1992). When individuals with such histories are later exposed to conflict-related violence, this may not only constitute a new traumatic event but also trigger reactivation of unresolved trauma (Pat-Horenczyk & Schiff, 2019; Goral et al., 2021). In such cases, anxiety may stem from both current threats and the re-emergence of earlier traumatic memories (Punamäki et al., 2019).

Although the psychological effects of conflict-related and sexual violence have each been studied, far less is known about their interaction. Limited research in Israel suggests that women with a history of gender-based violence experience greater psychological distress during conflict escalation (Sa’ar et al., 2011), but to our knowledge, no prior study has comprehensively examined the intersection of sexual violence, coping, resilience, and PTSD symptoms in this context. This study seeks to address that gap.

### 1.4. Theoretical Framework and Key Resilience Constructs

This study draws on an integrative theoretical model that combines the **socio-ecological theory**, the **transactional model of stress and coping**, and the **salutogenic theory** to understand how women cope with intersecting exposures to sexual and conflict-related violence. Each framework contributes uniquely to explaining how individuals perceive, process, and respond to cumulative trauma across personal and community levels.

**The socio-ecological theory** (Bronfenbrenner, 1979) views the individual within a broad context made up of several interconnected systems—such as family, community, and society—that shape perceptions, behavior, emotions, and the ability to successfully cope with distress. This study focuses on two personal-level (micro-system) factors (**sense of coherence [SOC] and coping strategies**) and one exo-system factor (**community resilience [CR]**), based on evidence that both personal and community-level resources significantly influence trauma outcomes (Ungar, 2011; Shapira et al., 2020; Stockman et al., 2023).

**The transactional model of stress and coping** (Lazarus & Folkman, 1984) emphasizes how individuals appraise threats and mobilize cognitive and behavioral strategies in response. Adaptive coping strategies (e.g., problem-solving) help mitigate distress by altering the individual–environment interaction, while non-adaptive strategies (e.g., avoidance, denial) are linked to poorer mental health outcomes, particularly in contexts of chronic threat (Albala & Shapira, 2024; Hirsch, 2022).

**The salutogenic model** (Antonovsky, 1979, 1987) complements these approaches by focusing on protective health factors, especially **sense of coherence (SOC)**—a worldview that life is comprehensible, manageable, and meaningful. A strong SOC has been linked to lower PTSD symptom severity in both conflict-related and sexual trauma contexts (Braun-Lewensohn & Mayer, 2020; Daneshvar et al., 2022; Dube & Rishi, 2017). While SOC supports adaptive coping, it may be weakened by early trauma, particularly when violence occurs within trusted relationships (Betke et al., 2021; Braun-Lewensohn et al., 2025).

To the best of our knowledge, the intersection of exposures to conflict-related and sexual violence and their relationship to SOC have not been explored previously. This study aims to address this gap in the literature.

At the **community level**, **perceived community resilience (CR)** reflects beliefs about the strength of the community and its vulnerabilities following experiences with potentially traumatic events (Shapira et al., 2020; Leykin et al., 2013). Perceived CR has been positively associated with both adaptive coping strategies (Korosteleva & Petrova, 2022) and levels of SOC (Mugisha, 2021). Higher CR has also been associated with lower psychological distress and greater coping capacity in both war and sexual violence contexts (Domhardt et al., 2015; Jaffe et al., 2022; Shapira et al., 2020). However, the role of CR in shaping post-traumatic symptoms among women exposed to both sexual and conflict-related violence has not yet been empirically tested.

### 1.5. Post-Traumatic Symptoms

According to the fifth edition of the *Diagnostic and Statistical Manual of Mental Disorders* (*DSM-5*), post-traumatic stress disorder (PTSD) is caused by direct or indirect exposure to an extreme event that poses a threat of death, serious injury, or harm to the physical integrity of the person or others. This disorder is characterized by symptoms of intrusion, avoidance of stimuli that remind the individual of the trauma, mood changes, and hyperarousal (American Psychiatric Association, 2022).

Sexual assault is one of the strongest risk factors for developing post-traumatic symptoms (Kaye-Tzadok & Davidson-Arad, 2016). In the context of conflict-related violence, PTSD responses tend to be more dynamic; unlike classic PTSD, symptoms often diminish once the threat subsides (Goral et al., 2021).

Coping strategies and resilience resources have been consistently linked to PTSD outcomes. Adaptive coping is negatively associated with post-traumatic symptoms, whereas non-adaptive strategies are positively associated with symptom severity, especially during ongoing conflict exposure (Cohen-Louck & Zvi, 2022; Shapira et al., 2025). Among survivors of sexual violence, adaptive coping similarly acts as a protective factor (Mahoney et al., 2021).

Sense of coherence (SOC) is negatively correlated with PTSD symptoms following both conflict-related (Kazlauskas et al., 2017) and sexual violence (Daneshvar et al., 2022). Community resilience (CR) has also been found to buffer against PTSD in conflict contexts (Shapira et al., 2020), yet its role in cases of sexual violence remains underexplored. To our knowledge, no prior study has examined how CR relates to PTSD symptoms among women with intersecting exposure to both conflict-related and sexual violence.

In sum, coping strategies, SOC, and CR are key factors shaping post-traumatic outcomes. However, their combined effects in cases of dual exposure have not yet been studied. This research seeks to fill that gap using an integrative model grounded in ecological, transactional, and salutogenic theories, with the aim of informing interventions to support women facing compounded trauma in conflict settings.

### 1.6. Research Questions and Hypotheses

1. **Do levels of coping strategies, SOC, CR, and post-traumatic symptoms differ in accordance with the level of exposure to conflict-related and/or sexual violence?**
  We hypothesized that significant group differences would be observed across the research variables. Women with high exposure to both sexual violence and conflict-related violence would be expected to report the highest use of non-adaptive coping strategies, the lowest levels of SOC and CR, and the most severe post-traumatic symptoms. In contrast, women with low exposure to both types of violence would be expected to report the lowest levels of non-adaptive coping strategies and post-traumatic symptoms, as well as the highest levels of adaptive coping, SOC, and CR.
2. **What are the relationships between exposure to ongoing conflict-related violence, exposure to sexual violence, coping strategies, SOC, CR, and post-traumatic symptoms?**
  We expected to find no correlation between the level of exposure to conflict-related violence and the level of exposure to sexual violence. We did expect to find positive correlations between the levels of exposure to conflict-related and sexual violence, the use of non-adaptive coping strategies, and the severity of post-traumatic symptoms. We also expected to find positive correlations between adaptive coping strategies, SOC, and CR. We expected to find negative correlations between levels of exposure and post-traumatic symptoms, on the one hand, and indicators of resilience (adaptive coping strategies, SOC, and CR) on the other.
3. **To what extent do combined exposure to ongoing conflict-related and sexual violence, sociodemographic characteristics (i.e., age and socioeconomic status), coping strategies, and resilience factors (i.e., SOC and CR) explain the severity of post-traumatic symptoms?**

## 2. Materials and Methods

### 2.1. Study Population and Sample

This study included 568 Jewish Israeli women, aged 18–81, of a broad range of socioeconomic statuses and varying levels of exposure to prolonged political violence and sexual violence. The decision to focus on Jewish Israeli women was made to ensure cultural and linguistic consistency in assessing highly sensitive experiences such as sexual and conflict-related violence. Israeli society is diverse, and the study describes the central ethnic group in Israel; therefore, the findings cannot be generalized to other ethnic or cultural populations.

Among the participants, the following was true:

- 104 women (18.3% of the sample) had been exposed to high levels of both sexual assault and conflict-related violence.
- 137 women (24.1% of the sample) had been exposed to high levels of conflict-related violence.
- 156 women (27.5% of the sample) had been exposed to high levels of sexual violence.
- 171 women (30.1% of the sample) had been exposed to low levels of both forms of violence.

The personal characteristics of the participants and the differences between the various groups in these characteristics are presented in Table 1.

### 2.2. Research Instruments

Given the sensitive and stigmatized nature of the study topic, data collection was conducted through an anonymous online questionnaire. This mode was chosen to minimize social desirability bias and reduce barriers to participation, especially among survivors of sexual violence, who may be less likely to disclose such experiences in face-to-face or clinical settings. The questionnaire included items for demographic characteristics, exposure to ongoing conflict-related violence, exposure to sexual violence, coping strategies, personal resilience, community resilience, and PTSD symptoms.

#### 2.2.1. Demographic Data

Data were collected regarding age, place of residence, education, marital status, employment status, and income level.

#### 2.2.2. Exposure to Ongoing Conflict-Related Violence

The intensity of exposure to ongoing conflict-related violence was measured using a structured questionnaire (Sagy & Braun-Lewensohn, 2009) consisting of nine items, which were each rated on a Likert scale ranging from 1 (*not at all*) to 4 (*very often* or *severely*). Six of these nine items referred to exposure to conflict-related events, including incendiary balloons/kites, rocket-fire, tunnel threats/suspected terrorist infiltrations, evacuation from one’s home, physical injury, and injury of a significant other. 

The exposure variable was analyzed in two ways. First, an average score of all items was calculated for each participant, creating a continuous variable representing exposure intensity. This variable was used in the structural-equation model. Then, a dichotomous exposure variable was created by calculating the sample median and classifying participants into high- or low-exposure groups based on whether their average score fell above or below the median. This classification was used to define the four exposure groups in the group comparison analyses.

#### 2.2.3. Exposure to Sexual Violence

The intensity of exposure to sexual violence was measured using a questionnaire developed for this study based on previous research scales (Koren-Karie et al., 2008). This questionnaire included 12 items, which were each rated on a 4-point Likert scale. Four of these items addressed the severity of the assault, such as exposure to genitalia, kissing, and penetration. Internal consistency for these four core items was acceptable (Cronbach’s alpha = 0.74).

Each item was weighted based on its severity: The item “Has someone inserted a body part or object into your body?” was given the highest weight. The items “Has someone kissed or rubbed against you against your will?” and “Has someone exposed you to sexual acts or pornographic material?” were given medium weight. The item “Has someone published a picture, recording, or video focusing on your sexuality without your consent?” was given the lowest weight. The sexual violence exposure scale was also analyzed in two ways. First, a continuous score was calculated by averaging the weighted items, representing overall exposure intensity. This variable was used in the structural equation model. Then, a dichotomous variable was created by calculating the sample median, classifying participants as high or low exposure. This variable was used to define the four exposure groups.

#### 2.2.4. Coping Strategies

Coping strategies were assessed using the Brief COPE inventory (Carver, 1997), a 28-item scale that measures responses to stressful events in terms of 14 distinct coping strategies: active coping, planning, positive reframing, acceptance, humor, religion, emotional support, instrumental support, distraction, denial, venting, substance use, behavioral disengagement, and self-blame. These strategies were grouped into two overarching categories: adaptive strategies and non-adaptive strategies (Carver, 1997). Each item was rated on a 4-point Likert scale ranging from 0 (*I usually do not do this*) to 3 (*I do this a lot*). Internal consistency (Cronbach’s alpha values) for the original subscales ranged from 0.50 to 0.92 (Carver, 1997). In the current study, the reliability of the questionnaire for the entire sample was α = 0.79 for adaptive coping strategies and α = 0.80 for the non-adaptive coping strategies.

#### 2.2.5. Personal Resilience

Personal resilience was measured using Antonovsky’s (1993) SOC scale, which measures three core components of SOC: comprehensibility, manageability, and meaningfulness. This study used the 13-item shortened version of this scale, which has demonstrated high correlation with the original scale (Antonovsky, 1993). The items were rated on a 7-point Likert scale, with higher scores indicating stronger SOC. In the current study, the internal consistency for the total sample was high (α = 0.89).

#### 2.2.6. Community Resilience (CR)

CR was assessed using the Conjoint Community Resilience Assessment Measure (CCRAM; Leykin et al., 2013), which evaluates individuals’ perceptions of their community, focusing on five factors: leadership, preparedness, collective efficacy, place attachment, and social trust. The current study employed the validated 10-item short version of this instrument, with responses rated on a 5-point Likert scale that ranged from 1 (*strongly disagree*) to 5 (*strongly agree*). For the current sample, the scale demonstrated excellent internal consistency (α = 0.91).

#### 2.2.7. Post-Traumatic Stress Disorder (PTSD)

PTSD was measured using the PTSD Checklist for *DSM-5* (PCL-5; Weathers et al., 2013), a 20-item, self-report measure aligned with *DSM-5* diagnostic criteria. This scale captures the presence and severity of symptoms from four symptom clusters: intrusion, avoidance, negative alterations in cognition and mood, and arousal. Each item is rated on a 5-point Likert scale ranging from 0 (*not at all*) to 4 (*extremely*), yielding a total score between 0 to 80, with higher scores indicating more severe symptoms. In the current study, the reliability for the total sample was α = 0.96.

### 2.3. Research Procedure

Following ethical approval from the Department of Conflict Resolution at Ben-Gurion University (Approval No. 2022-007, approval date: 9 July 2022), invitations to participate in the study were distributed through multiple channels. These included centers supporting women survivors of sexual violence across Israel and resilience centers in regional councils in the Gaza border area. In addition, the questionnaire was disseminated via social media and local distribution lists within these communities. Data were collected online using the Qualtrics platform. The introduction to the questionnaire provided an overview of the purpose of the study purpose and assured participants of full anonymity. Informed consent was obtained electronically before participants could access the full questionnaire.

### 2.4. Data Analysis

Data were coded and analyzed using SPSS (version 29). Scale reliabilities were assessed and descriptive statistics (frequencies, means, and standard deviations) were calculated for all study variables. Chi-square tests and *t*-tests were used to examine group differences in demographic variables. To assess differences in study variables across exposure groups, a one-way ANOVA was conducted, followed by Scheffé post hoc tests to identify the sources of significant differences. Pearson correlation coefficients were calculated to explore associations among the study variables. Finally, structural-equation modeling was conducted using AMOS (version 28) to evaluate the hypothesized relationships within an integrative model and to assess overall model fit.

## 3. Results

The means and standard deviations of the items from the conflict-related violence exposure questionnaire are presented in Table 2. Among the listed items, participants reported the highest exposure to rocket-fire. The corresponding data for the Sexual Violence Exposure questionnaire are presented in Table 3, with non-consensual physical contact, such as kissing or rubbing, identified as the most frequently reported experience.

### 3.1. Differences Between Research Groups in Terms of the Study Variables

The first research hypothesis was that women with dual exposure would report the highest use of non-adaptive coping strategies, the highest levels of post-traumatic symptoms, and the lowest levels of personal SOC, CR, and use of adaptive coping strategies. This hypothesis was partially confirmed. As hypothesized, the dual-exposure group reported the highest levels of non-adaptive coping strategies and post-traumatic symptoms, as well as the lowest level of SOC and the lowest use of adaptive coping strategies. However, contrary to the research hypothesis, the conflict-exposure group reported the highest levels of personal SOC and CR, as well as the lowest levels of non-adaptive coping strategies and post-traumatic symptoms. Also contrary to the hypothesis, the sexual-exposure group reported the highest levels of adaptive coping strategies and the lowest levels of CR.

**Figure A1:**
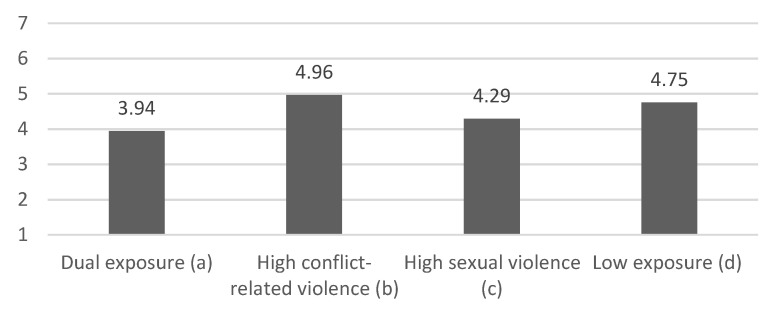
Differences in SOC levels across exposure groups. Each study group is labeled with a letter (a–d).

**Figure A2:**
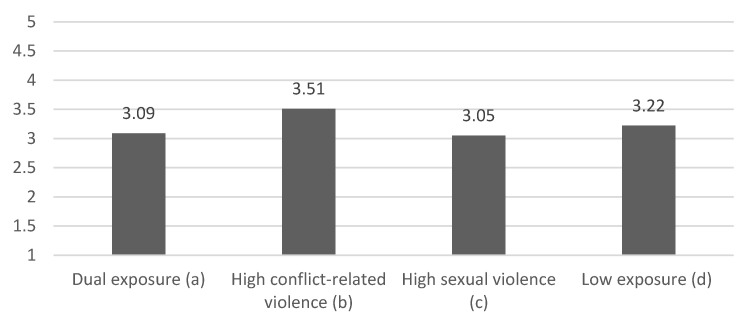
Differences in CR levels across exposure groups. Each study group is labeled with a letter (a–d).

**Figure A3:**
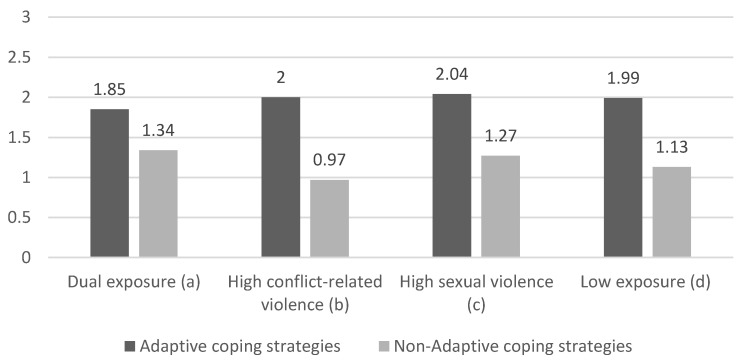
Differences in coping strategy levels across exposure groups. Each study group is labeled with a letter (a–d).

**Figure A4:**
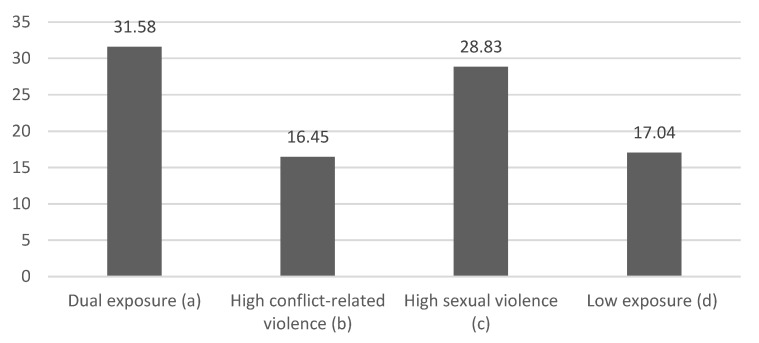
Differences in post-traumatic symptom levels across exposure groups. Each study group is labeled with a letter (a–d).

Most of the significant differences were between the dual-exposure group and the conflict-exposure group. Significant differences in the use of adaptive coping strategies were found between the dual-exposure group and the sexual-exposure group, with the sexual-exposure group reporting the highest use of adaptive coping strategies and the dual-exposure group reporting the lowest use of those strategies. These findings are presented in Table 4 and Figure A1, Figure A2, Figure A3 and Figure A4 in the Appendix A. For a detailed breakdown of group differences in the use of different coping strategies, please see Table A1 and Table A2 in the Appendix A.

### 3.2. Relationships Between Study Variables

To examine the second research hypothesis concerning the associations between the study variables, Pearson correlation tests were conducted. The findings from this analysis partially supported our hypothesis.

Consistent with our hypothesis, positive correlations were found between exposure to sexual violence and dual exposure, on the one hand, and post-traumatic symptom severity and non-adaptive coping strategies on the other. Negative correlations were found between exposure to sexual violence and dual exposure, on the one hand, and the use of adaptive coping strategies and the resilience resources SOC and CR on the other. Additionally, negative correlations were observed between the variables: use of adaptive coping strategies, SOC and CR, on the one hand, and the variables: use of non-adaptive coping strategies and post-traumatic symptom severity on the other.

As hypothesized, no correlation was found between the level of exposure to conflict-related violence and the level of exposure to sexual violence. Contrary to the research hypothesis, no significant correlations were found between conflict-related violence exposure and SOC, adaptive coping strategies, non-adaptive coping strategies, or post-traumatic symptom severity. However, unexpectedly, a positive correlation was found between exposure to conflict-related violence and CR (see Table 5).

### 3.3. Model Analysis

To synthesize the findings, a structural-equation model was constructed to examine the interplay among the study variables and their respective contributions to post-traumatic symptom severity among women in Israel. Exposure to conflict-related and sexual violence was measured as a cumulative variable. The model was estimated using AMOS software (version 29).

The model was evaluated using several fit indices, including the chi-square to degrees of freedom ratio (χ^2^/*df*), the Incremental Fit Index (IFI; Bollen, 1989), the Comparative Fit Index (CFI; Bentler, 1990), and the Root Mean Square Error of Approximation (RMSEA; Browne & Cudeck, 1993). Accepted thresholds for good model fit include a χ^2^/*df* ratio of 3 or lower (McIver & Carmines, 1981), IFI and CFI values of 0.90 or above, and an RMSEA value below 0.08 (Browne & Cudeck, 1993; Hoyle, 1995). The model fit the data very well, with χ^2^(4) = 5; *p* < 0.01; χ^2^/*df* = 1.26; CFI = 1.00; IFI = 1.00; TLI = 0.99; NFI = 1.00; and RMSEA = 0.02.

Only statistically significant paths were retained in the final model, with the exception of adaptive coping strategies, which were included to preserve theoretical consistency. The model explained 61% of the variance in post-traumatic symptoms. Among the direct predictors of post-traumatic symptoms, the combined exposure level (β = 0.20), socioeconomic status (β = 0.11), age (β = 0.07), SOC (β = −0.40), CR (β = −0.12), and non-adaptive coping strategies (β = 0.24) were significant, while adaptive coping strategies (β = −0.03) did not reach statistical significance. The strongest direct predictors—positively and negatively—were combined exposure, non-adaptive coping strategies, and SOC.

In terms of indirect effects, combined exposure level (β = 0.19), SOC (β = −0.20), socioeconomic status (β = −0.15), age (β = −0.14), and CR (β = −0.02) were all associated with post-traumatic symptoms, with combined exposure and SOC again emerging as the most influential factors. In addition, the direct effect of SOC on the use of non-adaptive coping strategies (−0.58) made an indirect contribution to the reduction in post-traumatic symptom levels. The direct effects between combined exposure level and SOC (−0.28) and between combined exposure level and the use of non-adaptive coping strategies (−0.07) indirectly contributed to increased post-traumatic symptoms.

**Figure 1:**
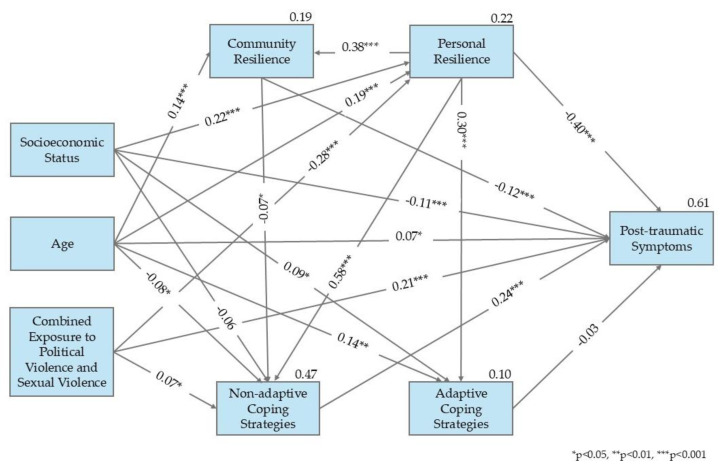
Research model.

An analysis of total effects confirmed these patterns, with combined exposure level (β = 0.40), SOC (β = −0.60), socioeconomic status (β = −0.26), non-adaptive coping strategies (β = 0.24), CR (β = −0.13), age (β = −0.07), and adaptive coping strategies (β = −0.03; non-significant) contributing to the overall model. These findings underscore the central role of combined trauma exposure, resilience resources, and coping strategies—particularly SOC and non-adaptive coping—in predicting the severity of post-traumatic symptoms (see Figure 1).

## 4. Discussion

This study aimed to examine how exposure to sexual and/or conflict-related violence—separately and cumulatively—relates to the severity of post-traumatic symptoms, and to assess the mediating roles of personal resources (i.e., SOC, coping strategies) and community resources (i.e., CR) in that relationship. This pioneering study is the first to examine dual exposure to both sexual violence and prolonged conflict-related violence through an integrative model grounded in ecological theory, the transactional model of coping with stress, and salutogenic theory. This theoretical framework offers a comprehensive understanding of how ecological and social factors shape individuals’ capacity to cope with chronic conflict-related stress (Shapira, 2022; Shapira et al., 2020) and prior sexual trauma (Stockman et al., 2023). By emphasizing the dynamic interaction between individuals and their environments, our model highlights the role of personal and community resources in shaping psychological outcomes (Lazarus & Folkman, 1984).

The first research question examined whether there were differences between the four exposure groups (i.e., exposure to sexual violence only, exposure to conflict-related violence only, dual exposure, and no exposure), in terms of the different study variables. This hypothesis was partially confirmed. Specifically, the dual-exposure group reported the highest levels of use of non-adaptive coping strategies and post-traumatic symptoms, as well as the lowest levels of SOC and use of adaptive coping strategies. This suggests that the accumulation of distinct types of trauma not only increases symptom severity, but also diminishes the individual’s capacity to utilize internal and external coping mechanisms (DeCandia & Guarino, 2015).

Regarding the use of adaptive coping strategies, the dual-exposure group reported the lowest levels of emotional support utilization, and those levels were significantly different from those observed for the high-sexual-violence group. This finding is consistent with research on resilience among survivors of sexual violence, which has highlighted high levels of reliance on emotional support resources (Domhardt et al., 2015). Furthermore, the dual-exposure group reported the lowest levels of use of active coping strategies, significantly differentiating that group from all of the other exposure groups. This finding illustrates the unique impact of intersecting stressful life events, including both acute traumatic events and chronic, prolonged stress, on coping mechanisms and the ability to seek emotional support from one’s environment (Nurius et al., 2013).

Moreover, the dual-exposure group reported the most use of non-adaptive coping strategies, such as denial, substance use, and avoidance strategies. One possible explanation for these findings is that, in the context of sexual violence, non-adaptive coping strategies, such as denial and avoidance, may provide temporary relief from negative emotions, leading to increased reliance on these strategies following exposure to additional traumatic events (Scarduzio et al., 2018). The findings of the current study are consistent with previous research indicating that non-adaptive coping strategies, particularly avoidance, serve as risk factors that contribute to heightened post-traumatic symptom severity in the context of both sexual and conflict-related violence (Boehm-Tabib & Gelkopf, 2021; Scarduzio et al., 2018). The literature further suggests that avoidant coping patterns increase distress by impairing the adaptation process, reducing awareness and knowledge, and preventing effective coping with stress (Herman, 1992). This emphasizes the clinical importance of identifying maladaptive coping strategies early, especially among women facing cumulative trauma, in order to provide targeted and effective treatment (Fernández-Fillol et al., 2021).

This finding aligns with previous research indicating that the intersection of stressful events creates unique and increased vulnerability compared to each event separately (Slopen et al., 2018). Specifically, dual exposure to both sexual and conflict-related violence leads to higher levels of distress than either form of exposure alone (Sachs et al., 2007). In this study, the combination of similar threats to bodily and psychological integrity, characterizing both prolonged conflict-related violence and sexual violence, was found to heighten vulnerability. This was reflected not only in elevated levels of post-traumatic symptoms, but also in increased use of non-adaptive coping strategies and lower levels of SOC, which emerged as key risk and protective factors in the integrative model, respectively.

Contrary to the research hypothesis, the conflict-related-violence exposure group reported the highest levels of SOC and CR, as well as the lowest levels of non-adaptive coping strategies and post-traumatic symptoms. This finding supports prior research showing that prolonged exposure to conflict-related violence does not necessarily lead to higher levels of post-traumatic symptoms, as compared to the general population (Greene et al., 2018). Levels of SOC among populations exposed to prolonged conflict-related violence are comparable to those reported in the general population, at least based on data collected before the October 7th attack (Baron-Epel et al., 2017).

This study also found a positive correlation between exposure to prolonged conflict-related violence and CR. This finding aligns with other research showing that communities exposed to prolonged conflict-related violence often exhibit higher-than-average CR, likely due to the presence of strong social support and collective resilience in such regions (Shapira, 2022). Extensive research in Israel and worldwide has demonstrated that CR helps individuals exposed to trauma to cope effectively with stress and threat (Zelis & Shapira, 2025) and serves as a protective factor against the effects of prolonged conflict-related violence (Braun-Lewensohn et al., 2025). In the current study, the conflict-related exposure group reported the highest levels of CR and the lowest levels of post-traumatic symptoms. A possible explanation for this is that the CR characterizing this group functions as a key protective factor, preventing the development of stress-related symptoms, such as post-traumatic symptoms. Notably, the dual-exposure group reported significantly lower levels of CR than the conflict-related-violence exposure group. This suggests that women who have experienced sexual violence, even if residing in conflict zones with available community resources, do not experience the same level of CR as women in those areas who have not experienced sexual violence. This may be due to stigma, internalized shame, or fractured trust in communal structures, often reported among sexual violence survivors (Wachter et al., 2018). Additionally, a negative correlation was found between exposure to sexual violence and CR, as well as between dual exposure and CR. These findings support the assumption that CR is less accessible to women who have been exposed to sexual violence, as compared to those who have not experienced such trauma. However, in the multivariate model, the contribution of community resilience was found to act primarily indirectly, through the use of non-adaptive coping strategies.

Analysis of the model, including an examination of direct, indirect, and total effects, indicates that the key variables explaining post-traumatic symptom severity are SOC, combined exposure to sexual and conflict-related violence, and the use of non-adaptive coping strategies. The model highlights the importance of ecological–social components in an individual’s ability to cope effectively with distress. This is consistent with the literature on coping with conflict-related violence (Shapira, 2022) and sexual violence (Stockman et al., 2023). Furthermore, these findings align with previous research that has emphasized the significance of salutogenic factors in these contexts (Dube & Rishi, 2017; Braun-Lewensohn & Mayer, 2020) and the crucial role of SOC in reducing distress in response to stressors, such as sexual violence (McGee et al., 2018) and prolonged conflict-related violence (Schäfer et al., 2019). Importantly, the integrative model enables a dynamic understanding not only of the outcomes, but also of the psychological and environmental processes through which they are formed.

Additionally, the model suggests that SOC contributes to reduced use of non-adaptive coping strategies and increased use of adaptive coping strategies. This finding supports prior research indicating a positive correlation between SOC and the use of adaptive coping strategies, as well as a negative correlation between SOC and the use of non-adaptive coping strategies (Solberg, 2021). Through SOC’s influence on coping strategies, it also indirectly enhances its negative effect on the severity of post-traumatic symptoms.

One of the unexpected findings was the non-significant direct effect of adaptive coping strategies on post-traumatic symptoms. A possible explanation for this result is a conceptual and statistical overlap between adaptive coping and the SOC construct, which itself strongly predicted lower PTSD symptoms. Since SOC captures individuals’ ability to perceive life events as comprehensible, manageable, and meaningful, it may have accounted for much of the variance that would otherwise be attributed to adaptive coping. Future studies could further explore this overlap to distinguish the unique contributions of each construct.

Moreover, the findings underscore differences in the contributions of ecological–social components from the micro-system (such as SOC and coping strategies), as compared to the exo-system (such as CR). It is evident that in the context of combined exposure to both prolonged conflict-related and sexual violence, the contribution of CR, as an exo-system component, is less significant than the contributions of the micro-system resources SOC and coping strategies. These differences may stem from disruptions in the interaction between ecological–social systems and the individual when transitioning from a stressor perceived as collective to one perceived as individually targeted.

### 4.1. Theoretical Contribution and Practical Implications

From a theoretical perspective, this study introduced and empirically tested, for the first time, an integrative framework that combines ecological theory, the transactional model of stress and coping, and salutogenic theory to explain the psychological impact of intersecting exposures to violence. By situating individual trauma within broader socio-environmental contexts, this study underscores the need to conceptualize resilience not only as an individual trait but also as a type of dynamic interplay between personal and community-level resources. This intersectional and multi-level approach contributes to a more nuanced understanding of how cumulative violence shapes mental health outcomes. It also expands theoretical models of trauma by integrating sociopolitical dimensions into ecological and salutogenic frameworks.

The primary contribution of this study to practice is the understanding that, in the context of PTSD symptom severity, the impact of exposure to both conflict-related violence and sexual violence is different from the impact of each type of exposure separately. This understanding highlights the importance of tailoring interventions to women who have been exposed to both of these types of violence.

Given the findings emphasizing the significance of socio-ecological factors and the substantial contribution of SOC and coping strategies in reducing PTSD symptoms among those who have been exposed to both conflict-related and sexual violence, an intervention program integrating individual, community, and systemic approaches is proposed. At the individual level, we recommend that efforts be made to develop a unique clinical model aimed at strengthening SOC, fostering adaptive coping strategies, and reducing non-adaptive coping strategies. Regarding the use of non-adaptive coping strategies, psychoeducational psychological work in clinical treatment settings can help individuals recognize these non-adaptive patterns, thereby enabling women with dual exposure to identify ineffective coping mechanisms and work toward adopting more adaptive strategies. Additionally, given the difference between women exposed solely to sexual violence and those exposed to both types of violence, specifically in the use of “emotional support” as a coping strategy, efforts should be made to improve the accessibility of welfare and mental health services for this population. Furthermore, mechanisms for identifying and detecting dual-exposure survivors should be developed.

At the community level, our findings highlight the importance of how dual-exposure survivors perceive their community, revealing that individuals with dual exposure may experience a diminished capacity to draw on the community as a source of resilience. Therefore, it is recommended that professionals in local communities invest efforts in strengthening the connection between these survivors and their communities in two ways. First, by expanding community awareness regarding the intersection of conflict-related and sexual violence exposure, as well as the unique characteristics and needs of dual-exposure survivors. Second, by assisting survivors in connecting with personal and social support networks. Group-based interventions that combine peer support with professional facilitation may be particularly effective in rebuilding trust and fostering CR.

At the systemic policy level, it is essential to implement trauma-informed approaches across sectors that routinely engage with survivors, including healthcare, education, and social services. In conflict-affected areas, community-based mental health infrastructure must be expanded to ensure accessible, sustained support. Early identification and screening programs should be established in primary care and community settings to detect dual exposure and facilitate timely referrals. To ensure effective, coordinated care, intersectoral collaboration between mental health providers, local authorities, women’s organizations, and resilience centers should be formally institutionalized.

### 4.2. Limitations of the Study

This study has several limitations. The first limitation is that the data were collected between December 2022 and January 2023, during a period of relative calm. It can be assumed that the collected data do not fully represent the situation during more turbulent conflict-related violence periods, particularly in the aftermath of the terrorist attack on 7 October 2023, and during the subsequent war. However, this context also provides a unique opportunity to examine coping mechanisms and resilience during a prolonged state of insecurity, offering a valuable baseline for future research conducted under conditions of acute threat. Stress and trauma responses are known to be dynamic and context-sensitive, shaped by the severity and duration of threat as well as broader socio-political conditions (Muldoon et al., 2021). Future longitudinal research could assess how the escalation of violence affects psychological symptoms, disrupts coping processes, or challenges resilience resources, particularly among survivors of sexual violence.

Second, the study’s cross-sectional design limits the ability to draw causal inferences or assess changes over time. To obtain a broader and more accurate understanding of the effects of dual exposure, future research should incorporate longitudinal data collection across multiple time points and varying levels of conflict intensity.

Third, the measurement of the intensity of exposure to sexual violence. This was assessed using a questionnaire designed for this study based on previously used research instruments. While the items were carefully selected to reflect different forms of sexual violence and showed acceptable internal consistency, the tool has not undergone external validation. This limits comparability across studies and may affect the generalizability of findings. However, incorporating additional characteristics of sexual violence—such as the age at which the assault occurred, its duration, the perpetrator’s identity, and other contextual factors—could provide a more comprehensive understanding of the severity and impact of the experience. Therefore, the findings should be interpreted with an awareness of this partial representation of exposure characteristics.

A fourth limitation relates to the study’s reliance on self-report instruments to assess highly sensitive experiences, including exposure to sexual violence. Such measures are inherently susceptible to recall and disclosure biases, particularly in contexts where stigma, shame, or trauma-related avoidance may impact participants’ willingness or ability to report accurately. Additionally, the use of a structured, anonymous online questionnaire and convenience sampling may introduce sampling bias and limit representativeness. However, this approach was ethically and practically justified: anonymous self-report is commonly used in trauma research to reduce barriers to disclosure and protect participant well-being, especially when addressing stigmatized or potentially retraumatizing experiences (Jaffe et al., 2015). While the instruments used were based on validated tools and adapted with input from clinical experts, they were not externally validated within the specific context of this study. This limitation should be considered when interpreting the findings, and future research would benefit from incorporating multiple methods of assessment, including clinician-administered tools or behavioral data, where appropriate and ethically feasible.

And finally, this study focused exclusively on Jewish Israeli women, the central ethnic group in Israel. Therefore, caution should be exercised when generalizing the findings to other populations. The Israeli society is diverse, and the findings may not be applicable to other cultural groups, especially in contexts of intersecting violence, coping processes, and resilience resources (Abu-Raiya et al., 2020; Baron-Epel et al., 2017). The decision to focus on Jewish Israeli women was driven by ethical, methodological, and logistical considerations. In a context of ongoing conflict, recruiting participants from across ethnic and national lines poses significant ethical and safety challenges, particularly when addressing highly sensitive topics such as sexual violence. We fully acknowledge that other groups—such as Palestinian women, both within Israel and in the occupied Palestinian territories—are affected by intersecting violence in different ways. Future research should prioritize inclusive and comparative designs to understand how cultural, geopolitical, and community factors may influence resilience, coping strategies, and post-traumatic symptoms in the context of combined exposure to violence.

To conclude, this study provides novel empirical evidence regarding the psychological consequences of dual exposure to sexual violence and prolonged conflict-related violence among women. The findings reveal that women who have experienced both these types of violence report significantly higher levels of post-traumatic stress symptoms and lower resilience indicators, as compared to those who have experienced either type of violence alone. This suggests that the cumulative effect of multiple, intersecting threats produces a distinct and intensified trauma response.

This study also highlights the critical roles of personal and community resources in mitigating these effects. In particular, SOC emerged as a key protective factor, reducing PTSD symptom severity directly and also indirectly through its influence on coping strategies. These findings reinforce the value of integrating ecological, salutogenic, and coping-based frameworks when examining trauma in conflict-affected populations.

The results of this work call attention to the need for trauma-informed, multi-level interventions that recognize the compounded vulnerability of women exposed to intersecting forms of violence. Strengthening SOC and enhancing community resilience should be central components of such efforts. Future research should build on these findings by employing longitudinal designs and examining diverse cultural and geopolitical contexts to further understand how intersecting exposures shape trauma trajectories and recovery processes.

## 5. Conclusions

This study provides novel empirical evidence regarding the psychological consequences of dual exposure to sexual violence and prolonged conflict-related violence among women. The findings reveal that women who have been exposed to both forms of violence report significantly higher levels of post-traumatic stress symptoms and lower resilience indicators, as compared to those exposed to either form of violence alone. This suggests that the cumulative effect of multiple, intersecting threats produces a distinct and intensified trauma response.

This study also highlights the critical role of personal and community resources in mitigating these effects. In particular, SOC emerged as a key protective factor, both directly reducing PTSD symptom severity and indirectly doing so through its influence on coping strategies. These findings reinforce the value of integrating ecological, salutogenic, and coping-based frameworks when examining trauma in conflict-affected populations.

From a theoretical perspective, this study is the first to empirically test an integrative framework combining ecological theory, the transactional model of stress and coping, and salutogenic theory to explain the impact of intersecting violence exposures. It reconceptualizes resilience as a dynamic interaction between individual and community-level resources, offering a more nuanced, multi-level understanding of how cumulative trauma shapes mental health outcomes.

From a practical perspective, the results call attention to the need for trauma-informed, multi-level interventions that recognize the compounded vulnerability of women exposed to intersecting forms of violence. Strengthening SOC and enhancing community resilience should be central components of such efforts.

Future research should build on these findings by employing longitudinal designs and examining diverse cultural and geopolitical contexts to further understand how intersecting exposures shape trauma trajectories and recovery processes.

## Figures and Tables

**Table 1:** Personal characteristics.

Variable		The Entire Sample *n* = 568	Dual Low Exposure *n* ≈ 171	High Exposure to Sexual Violence *n* ≈ 156	High Exposure to Conflict-Related Violence *n* ≈ 137	Dual High Exposure *n* ≈ 104	χ^2^/*F*
			*n* (%)	*n* (%)	*n* (%)	*n* (%)	
Age (*M* ± *SD*)	18–81	41.44 ± 12.99	40.29 ± 12.3	41.3 ± 12.48	43.28 ± 13.82	43.28 ± 13.68	1.27
Marital status	In a relationship	406 (71)	127 (73.8)	99 (63.5)	109 (79)	71 (67)	9.66 *
	Not in a relationship	166 (29)	45 (26.2)	57 (36.5)	29 (21)	35 (33)	
Level of religiosity	Secular	361 (65.75)	100 (60.24)	110 (72.36)	84 (63.63)	67 (67.67)	5.61
	Traditional/religious/ultra-orthodox	188 (34.24)	67 (39.75)	42 (27.63)	48 (36.36)	32 (32.32)	
Education	Academic	422 (75.3)	134 (80.2)	109 (71.2)	101 (74.8)	75 (73.5)	3.72
	Not academic	138 (24.6)	33 (19.8)	44 (28.8)	34 (25.2)	27 (26.5)	
Income level	Below average	282 (49.3)	76 (44.4)	77 (49.4)	63 (46)	66 (63.5)	13.1 *
	Average or close to average	140 (24.5)	48 (28.1)	38 (24.4)	40 (29.2)	14 (13.5)	
	Above average	146 (25.5)	47 (27.5)	41 (26.3)	34 (24.85)	24 (23.1)	
Employment	Unemployed	71 (13.2)	21 (13)	20 (12.8)	11 (8)	19 (17)	6.48
	Temporary jobs/part-time job	147 (27.3)	48 (29.8)	37 (23.7)	35 (25.4)	27 (25.4)	
	Full-time job/freelancer	320 (59.5)	92 (57.1)	86 (55.1)	86 (62.3)	56 (52.8)	

* *p* < 0.05.

**Table 2:** Exposure to ongoing conflict-related violence (scale range: 1–4).

Item	Mean	*SD*
Incendiary balloons/kites	1.66	1.09
Rocket-fire	3.00	1.09
Tunnel threat or fear of terrorist infiltration	1.69	1.02
Evacuation from home due to conflict-related threat	1.69	1.00
Physical injury	1.05	0.31
Injury of someone who is close to the participant	1.50	0.87

**Table 3:** Measures of exposure to sexual violence (range: 1–4).

Item	Mean	*SD*
Has anyone kissed or touched you in a sexual manner without your consent?	2.44	0.97
Has anyone exposed their genitals, sexual acts, or any form of pornographic material to you?	1.92	1.01
Has anyone published a picture, recording, or video focusing on your sexuality without your consent?	1.06	0.31
Has anyone inserted a body part or object into your genitals, anus, or mouth without your consent?	1.43	0.84

**Table 4:** Differences between exposure groups, in terms of the study variables.

Variable	Scale Range	Low Exposure (d) *n* ≈ 165		High Sexual Violence (c) *n* ≈ 156		High Conflict-Related Violence (b) *n* ≈ 135		Dual Exposure (a) *n* ≈ 104		Cohen’s *d*	*F* ^#^
		*SD*	*M*	*SD*	*M*	*SD*	*M*	*SD*	*M*		
SOC	1–7	1.01	4.75	1.24	4.29	1.03	4.96	1.33	3.94	0.79 ^ab^ 0.68 ^ad^ 0.59 ^bc^ 0.40 ^cd^	19.72 ^ab,ad,bc,cd^ **
CR	1–5	0.82	3.22	0.8	3.05	0.8	3.51	0.84	3.09	0.55 ^ab^ 0.62 ^bc^ 0.38 ^bd^	9.3 ^ab,bc,bd^ **
Adaptive coping strategies	0–3	0.45	1.99	0.5	2.04	0.54	2	0.53	1.85	0.36 ^ac^	3.03 ^ac^ *
Non-adaptive coping strategies	0–3	0.46	1.13	0.55	1.27	0.47	0.97	0.62	1.34	0.67 ^ab^ 0.4 ^ad^ 0.58 ^bc^	12.48 ^ab,ad,bc^ **
Post-traumatic symptoms	0–80	14.37	17.04	18.64	28.83	13.76	16.45	18.43	31.58	0.40 ^ab^ 0.88 ^ad^ 0.75 ^bc^ 0.70 ^cd^	31.39 ^ab,ad,bc,cd^ **

Note. CR—community resilience; SOC—sense of coherence. ^#^ Each study group is labeled with a letter (a–d); significant pairwise differences (*p* < 0.05 or *p* < 0.001) are denoted by combinations of these letters (e.g., ‘ab’ indicates a significant difference between groups a and b). * *p* < 0.05; ** *p* < 0.001.

**Table 5:** Pearson correlations between study variables (*N* ≈ 542).

Variable	Sexual Violence Exposure	Conflict-Related Violence Exposure	Combined Exposure to Conflict-Related Violence and Sexual Violence	SOC	CR	Adaptive Coping Strategies	Non-Adaptive Coping Strategies	PTSD Symptoms
Sexual violence exposure	1							
Conflict-related violence exposure	0.16−	1						
Combined exposure to conflict-related violence and sexual violence	0.90 **	0.40 **	1					
SOC	−0.38 **	0.009	−0.34 **	1				
CR	−0.21 **	0.16 **	−0.13 **	0.41 **	1			
Adaptive coping strategies	−0.10 *	−0.03	−0.10 *	0.29 **	0.16 **	1		
Non-adaptive coping strategies	0.36 **	−0.07	0.29 **	−0.67 **	−0.33 **	−0.09 *	1	
PTSD symptoms	0.50 **	−0.005	0.44 **	−0.71 **	−0.39 **	−0.22 **	0.62 **	1

Note. PTSD—post-traumatic stress disorder. * *p* < 0.05; ** *p* < 0.001.

## Data Availability

The data collected and used in this research are available from the authors upon reasonable request.

## Abbreviations

The following abbreviations are used in this manuscript:

CR	Community resilience
PTSD	Post-traumatic stress disorder
SOC	Sense of coherence

## Appendix A

**Table A1:** Differences between groups, in terms of the use of different adaptive coping strategies.

Variable	Low Exposure (d) *n* ≈ 165		High Sexual Violence (c) *n* ≈ 156		High Conflict-Related Violence (b) *n* ≈ 135		Dual Exposure (a) *n* ≈ 104		Cohen’s *d*	*F* ^#^
	*SD*	*M*	*SD*	*M*	*SD*	*M*	*SD*	*M*		
Active coping	0.75	2.16	0.75	2.17	0.75	2.2	0.84	1.88	0.4 ^ab^ 0.36 ^ac^ 0.35 ^ad^	4.3 ^ab,ac,ad^ *
Emotional support	0.82	2.12	0.83	2.14	0.86	2.09	0.86	1.81	0.39 ^ac^ 0.36 ^ad^	3.89 ^ac,ad^ *
Instrumental support	0.82	1.71	0.88	1.54	0.81	1.53	0.83	1.62		1.65
Positive reframing	0.78	1.91	0.82	1.94	0.79	1.98	0.88	1.85		0.55
Planning	0.62	2.4	0.7	2.41	0.68	2.35	0.76	2.18		2.95 *
Acceptance	0.66	1.71	0.74	1.86	0.78	1.83	0.79	1.88		1.55

Note. ^#^ Each study group is labeled with a letter (a–d); significant pairwise differences (* *p* < 0.05) are denoted by combinations of these letters (e.g., ‘ab’ indicates a significant difference between groups a and b).

**Table A2:** Differences between groups, in terms of the use of different non-adaptive coping strategies.

Variable	Low Exposure (d) *n* ≈ 165		High Sexual Violence (c) *n* ≈ 156		High Conflict-Related Violence (b) *n ≈* 135		Dual Exposure (a) *n* ≈ 104		Cohen’s *d*	*F* ^#^
	*SD*	*M*	*SD*	*M*	*SD*	*M*	*SD*	*M*		
Distraction	0.73	1.52	0.75	1.51	0.69	1.47	0.78	1.63		0.99
Denial	0.63	0.63	0.79	0.76	0.61	0.44	0.88	1.93	1.96 ^ab^ 1.39 ^ac^ 1.69 ^ad^ 0.45 ^bc^	10.3 ^ab,ac,ad,bc^ **
Substance use	0.8	0.43	0.95	0.65	0.67	1.31	1.07	0.79	0.58 ^ab^ 0.38 ^ad^ 0.80 ^bc^	7.59 ^ab,ad,bc^ **
Avoidance	0.78	1	0.77	1.04	0.68	0.88	0.71	1.13	0.35 ^ab^	2.81 ^ab^ *
Venting	0.8	1.64	0.84	1.88	0.89	1.35	0.88	1.75	0.45 ^ab^ 0.61 ^bc^ 0.34 ^bd^	9.91 ^ab,bc,bd^ **
Self-blame	0.9	1.54	0.99	1.74	0.9	1.34	0.97	1.8	0.49 ^ab^ 0.42 ^bc^	6.25 ^ab,bc^ **

Note. ^#^ Each study group is labeled with a letter (a–d); significant pairwise differences (* *p* < 0.05 or ** *p* < 0.001) are denoted by combinations of these letters (e.g., ‘ab’ indicates a significant difference between groups a and b).
